# Dataset of mechanical, marshall and rheological properties of crumb rubber – Bio-oil modified hot mix asphalt for sustainable pavement works

**DOI:** 10.1016/j.dib.2018.09.080

**Published:** 2018-09-29

**Authors:** Abayomi Emmanuel Modupe, Olumoyewa Dotun Atoyebi, Opeyemi Emmanuel Oluwatuyi, Oluwasegun James Aladegboye, Ayobami Adebola Busari, Adebayo Ofonime Basorun

**Affiliations:** a Department of Civil Engineering, Landmark University, Omu-Aran, Nigeria; b Department of Civil Engineering, Covenant University, Canaan Land, Ota, Nigeria

## Abstract

This data article presents information on the modification of bitumen with bio-oil pyrolyzed from cassava peels, and upgraded with a non-degradable polymer i.e. crumb rubber. Performance tests were carried out on the bio-oil crumb rubber modified bitumen. The main objective of bitumen modification is to produce new binders with improved mechanical, marshall and rheological properties [1]. The percentage of bio-oil by volume used for modification of bitumen was 5%, 10%, 15% and 20% respectively. Marshall Stability and flow tests were also carried out on the crumb rubber bio-asphalt produced.

**Specifications Table**

**Table:** 

Subject area	Civil engineering
More specific subject area	Transportation Engineering and Highway Materials, Sustainable Pavement Engineering and Design
Type of data	Table, image, graph, figure
How data was acquired	Production of Bio – Oil using a fabricated pyrolyzer of internal diameter 30 cm, Radius 15 cm, Thickness 2 cm and Height 41 cm as shown in Fig. 2 and conducting laboratory experiments on control and modified samples.
Data format	Raw
Experimental factors	Bio – Oil was Produced from Cassava Peels as studied in previous literature [2] and characterized to determine suitability as replacement for conventional binder. They were washed to remove lateritic impurities and dried in an oven.
Experimental features	Bio – Oil produced was upgraded by blending it with a polymer [3], [4] crumb rubber (from waste scrap tyres) in order to improve its mechanical properties and subsequently bitumen.
Data source location	Landmark University Highway and Geotechnical Engineering Laboratory, Omu Aran, Kwara State, Nigeria.
Data accessibility	Data is presented in this article.
Related research article	Mohamed Metwally, Mohamed Abdel Raouf and Williams, R. Christopher. (2010). *"Development of Non-Petroleum Based Binders for Use in Flexible Pavements.* Iowa State University [4].

**Value of the data**

• Data in this article can be used for the design of sustainable flexible pavement structure incorporating bio-oil and crumb rubber. Bio-oil can be utilized to produce bio-asphalt by using it to modify petroleum asphalt [5].
• Data obtained can be used for the planning and development of a bio-based economy.
• Data presented here under could be helpful in further research on bio-oil and crumb rubber modification of bitumen. It would also be relevant as it gives information on the possibility of conserving our natural mineral resources by recycling non degradable wastes and applying them in the road construction industry and at the same time reducing environmental pollution triggered by them.

## 1 Data

**Fig. 1:**
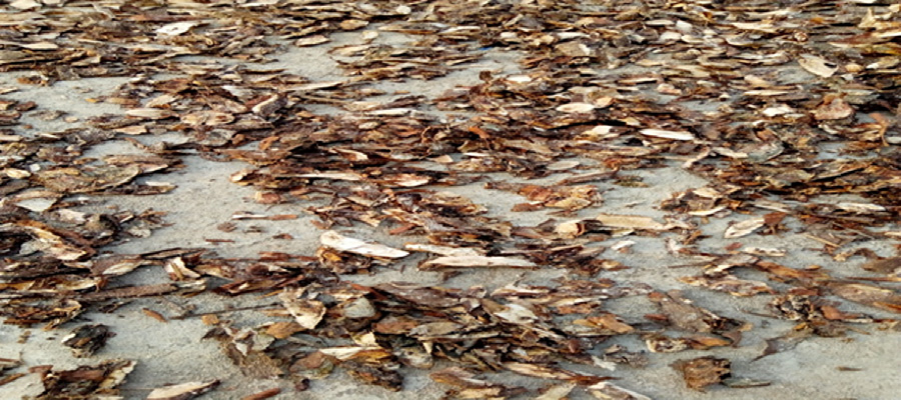
Cassava peels.

**Fig. 2:**
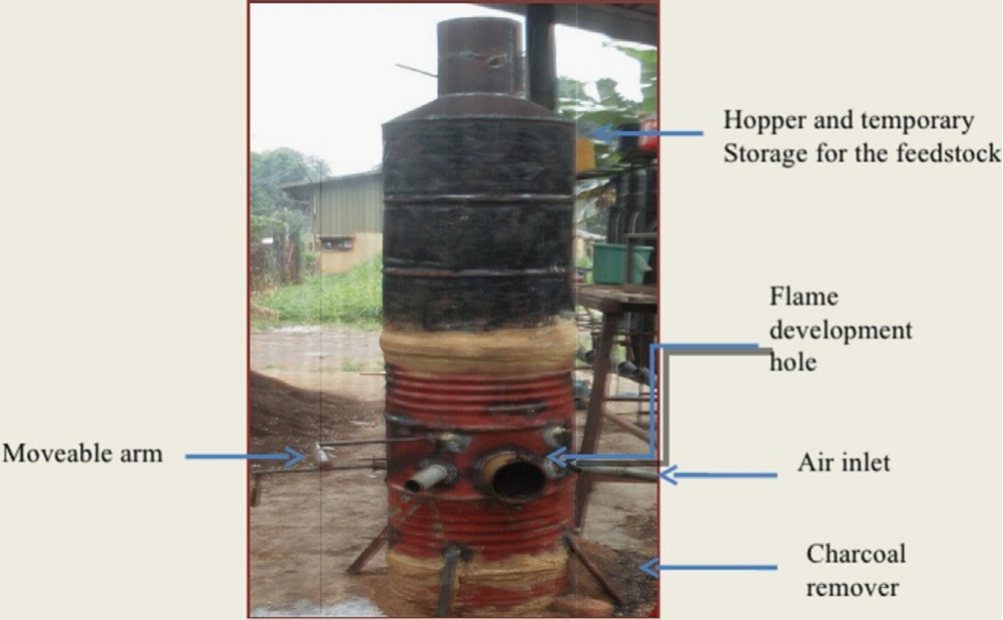
Fabricated pyrolyzer.

**Fig. 3:**
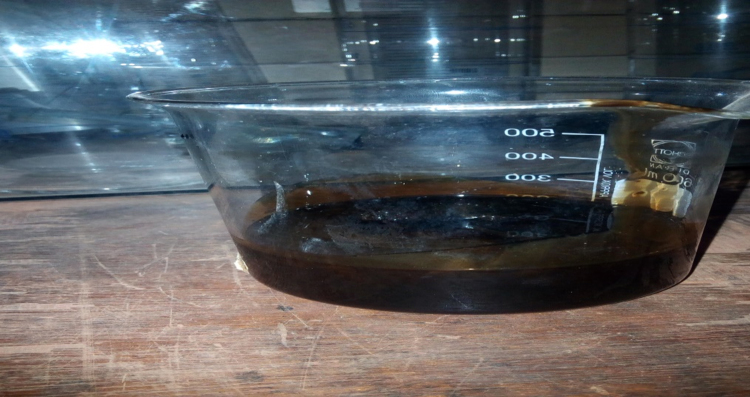
Sample of bio-oil extracted from cassava peels by pyrolysis.

The dataset represents the experimental results of penetration, flash point, fire point, softening point, specific gravity, moisture content viscosity and ductility of virgin bitumen and bitumen modified with bio-oil from cassava peels and upgraded with crumb rubber [6], [7]. It also reveals the particle size distribution of mineral aggregates used and the outcomes of Marshall Stability and flow test conducted on the polymer bio-asphalt subsequently produced. Laboratory tests were conducted at the Highway and Geotechnical Engineering Laboratory of Landmark University. Fig. 1 shows the bio mass (cassava peels) used in the investigation and the pyrolyzer fabricated to produce bio-oil from it on a small scale is as shown in Fig. 2. Fig. 3 shows the bio-oil extracted from the cassava peels. Table 1 shows the elemental composition of bio oil compared with virgin bitumen. The Effect of blending bio-oil and polymer on penetration grade of bitumen is shown in Table 2. Table 3 presents the influence of bio-oil modification on the ductility of bitumen. Effects of modification on Softening Point of bitumen are shown on Table 4. On Table 5, Table 6, Table 7, Table 8, Table 9 are results depicting the effect of modification on loss on heating, specific gravity, moisture content, flash & fire point, and viscosity of bitumen respectively. Table 10, Table 11, Table 12, Table 13 are results of coarse aggregate characterization, particle size distribution, marshall properties for bio-oil modification, and marshall properties for bio-oil & crumb rubber modification respectively.

**Table 1:** Elemental Analysis of Bio-oil compared to virgin bitumen.

Elemental composition (%)	Cassava peel bio-oil	Bitumen
Carbon	57	85
Hydrogen	4.85	11
Oxygen	38	1
Nitrogen	0.15	0.3

**Table 2:** Effect of bio-oil and Crumb rubber on penetration grade of bitumen.

Bio-oil percentage by volume of Bitumen	Penetration value (mm)		Penetration grade	
	Bio-oil	Bio-oil + Crumb rubber	Bio-oil	Bio-oil + Crumb rubber
0% (Control)	67	67	60/70	60/70
5%	62	61	60/70	60/70
10%	76	54	70/80	50/60
15%	81	51	80/90	50/60
20%	93	47	80/100	40/50

**Table 3:** Effects of bio-oil modification on ductility of bitumen.

Bio-oil percentage by volume of bitumen	Ductility (cm)		
	Bio-oil	Bio-oil + Crumb rubber	Standard requirement (Minimum)
0% (Control)	80	80	75
5%	82.3	83.3	75
10%	71	84.7	75
15%	69	87.2	75
20%	55	87.9	75

**Table 4:** Effects of bio-oil modification on softening point of bitumen.

Bio-oil percentage by volume ofbitumen	Softening point (°C)		
	Bio-oil	Bio-oil +Crumb rubber	Standard requirements
0% (Control)	54.5	54.5	45–60
5%	55	57.5	45–60
10%	55.5	58.5	45–60
15%	56.5	62	45–60
20%	57.5	62.5	55–65

**Table 5:** Effect of bio-oil modification on the loss on heating of bitumen.

Bio-oil percentage by volume of bitumen	Loss on heating (%)	
	Bio-oil	Bio-oil + Crumb rubber
0% (Control)	0.88	0.88
5%	0.92	0.89
10%	0.96	0.81
15%	1.02	1.18
20%	1.18	1.39

**Table 6:** Effect of bio-oil modification on specific gravity of bitumen.

Bio-oil percentage by volume of bitumen	Specific gravity		
	Bio-oil	Bio-oil + Crumb rubber	Standard requirement
0%	0.98	0.98	0.96–1.02
5%	0.973	1.03	0.96–1.02
10%	0.991	0.98	0.96–1.02
15%	1.012	0.97	0.96–1.02
20%	1.03	0.96	0.96–1.02

**Table 7:** Effect of bio-oil modification on the moisture content of bitumen.

Bio-oil percentage by volume of bitumen	Moisture content (%)	
	Bio-oil	Bio-oil + Crumb rubber
0%	0.09	0.09
5%	0.11	0.1
10%	0.19	0.14
15%	0.21	0.16
20%	0.29	0.19

**Table 8:** Effect of bio-oil modification on the flash & fire point of bitumen.

Bio-oil percentage by volume of bitumen	Flash point (°C)			Fire point (°C)		
	Bio-oil	Bio-oil + Crumb rubber	Standard requirement	Bio-oil	Bio-oil + Crumb rubber	Standard requirement
0%	240	250	175	240	250	205
5%	244	252	175	259	266	205
10%	250	267	175	265	274	205
15%	252	270	175	269	283	205
20%	259	271	175	274	`287	205

**Table 9:** Effect of bio-oil modification on the viscosity of bitumen.

Bio-oil percentage by volume of bitumen	Viscosity (s)	
	Bio-oil	Bio-oil + Crumb rubber
0%	275	275
5%	275	278
10%	272	284
15%	270	291
20%	269	298

**Table 10:** Coarse aggregate characterization.

Tests carried out	Test results obtained	Standard test values
Aggregate Impact Test	24.98%	30% Maximum
Aggregate Crushing Test	44.93%	45% Maximum
Los Angeles Abrasion Test	56.03	60% Maximum
Flakiness Index	28.62	30% Maximum
Elongation Index	29.53	30% Maximum
Density	1492.267 kg/m^3^	1500 kg/m^3^
Specific Gravity	2.8	2.8

**Table 11:** Particle size distribution for coarse aggregates.

Sieve no (#)	Sieve size (mm)	Weight of aggregates retained (g)	% retained on each sieve	Cumulative % retained on each sieve	Cumulative % passing
	
3/4	19	0	0	0	100
1/2	12.7	526	28.56	28.56	71.44
3/8	9.52	375	20.36	48.92	51.08
4	4.75	155	8.41	57.33	42.67
10	2	234	12.7	70.03	29.97
16	1.18	95	5.16	75.19	24.81
30	0.6	175	9.5	84.69	15.31
40	0.425	181	9.83	94.52	5.48
50	0.3	44.5	2.42	96.94	3.06

**Table 12:** Marshall properties for bio-oil modification.

%	PMB	Stability	Flow (mm)	*V*_v_	*V*_b_	*V*_fb_	*V*_ma_	*G*_m_	OBC (%)
Bio-oil									
0%	–	–	–	–	–	–	–	–	–
5%	Bio-Oil	12.5	10.67	3.8	17	81.9	20.7	2.3	5.7
10%	Bio-Oil	12	10.83	3.9	17	81.3	20.9	2.3	5.2
15%	Bio-Oil	15.67	11.17	4.5	16.9	79	21.3	2.3	5.6
20%	Bio-Oil	19	11.5	4.4	16.9	79.5	21.2	2.3	5.6

**Table 13:** Marshall properties for bio-oil & crumb rubber modification.

%	PMB	Stability	Flow (mm)	*V*_v_	*V*_b_	*V*_fb_	*V*_ma_	*G*_m_	OBC (%)
Bio-oil									
0	–	–	–	–	–	–	–	–	–
5	Bio-oil + Crumb rubber	19.33	11.17	4	16.9	79.4	21.3	2.3	5.4
10	Bio-oil + Crumb rubber	21.33	11.33	3.7	17	82.2	20.7	2.3	5.9
15	Bio-oil + Crumb rubber	25.67	12.83	4.1	16.9	80.4	21	2.3	5.1
20	Bio-oil + Crumb rubber	26.5	13.33	3.3	17	83.8	20.4	2.3	5.1

## 2 Experimental design, materials, and methods

**Fig. 4:**
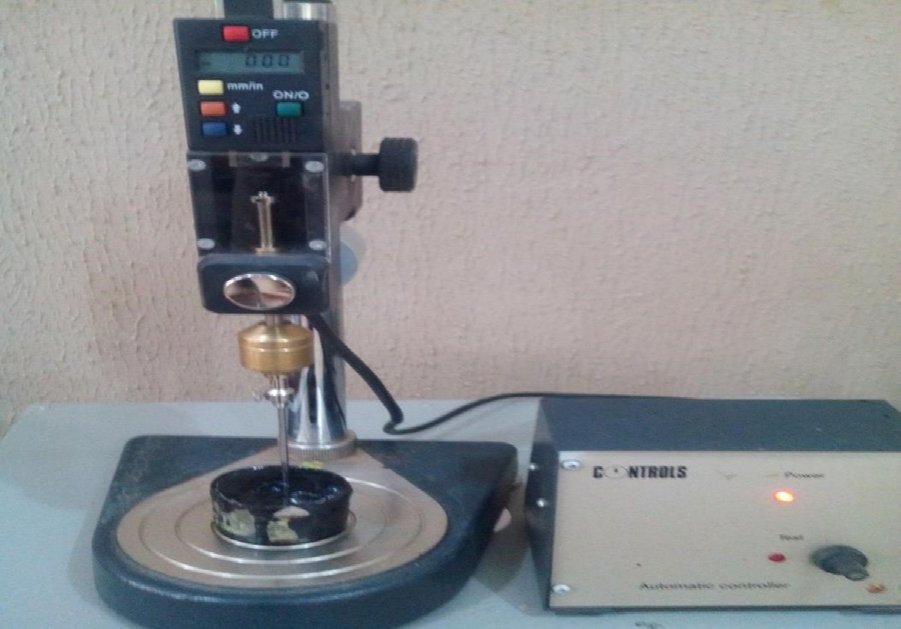
Penetration apparatus.

**Fig. 5:**
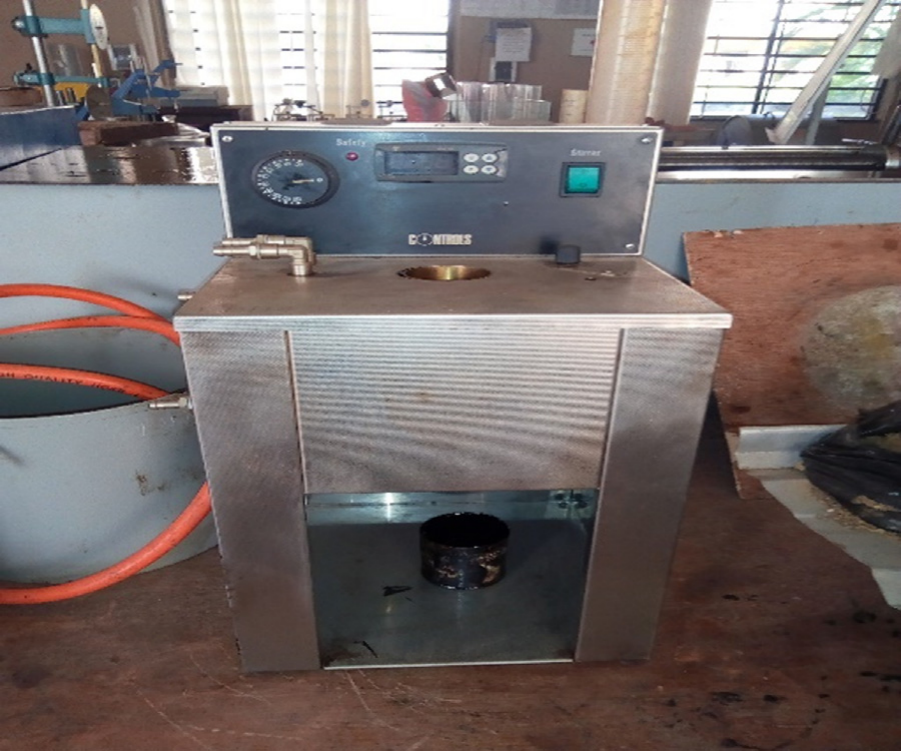
Viscometer.

The biomass (cassava peels) was sourced from the *Garri* processing plant located at the commercial farm of Landmark University, Omu-Aran, Kwara State, Nigeria. The bitumen was obtained from a bitumen processing plant in Akure, Ondo State. The mineral aggregates used for the production of bituminous concrete were sourced from Omu-aran, Kwara state, Nigeria. Crumb rubber used as polymer was sourced from the scrap dump site of the Physical Planning Department of Landmark University. The Crumb Rubber was milled to powder [8], the proportion divided for modification was the % passing sieve no 200 mm diameter mesh. The bio oil was extracted by pyrolysis, which involved the combustion of the dried cassava peels at a temperature of 529 °C, in an anaerobic condition i.e. in the absence of oxygen and consequently produced solid (bio char), bio-oil, and bio gas. Bitumen was blended with the milled crumb rubber using a high speed shear emulsifying machine at 180 °C [9], at 5%, 10%, 15% and 20% respectively and subsequently bio-oil was added to the mixture. The modified samples produced were subjected to penetration, density, ductility, flash and fire point, viscosity, loss on heating, softening point, specific gravity tests and water content tests using the appropriate testing equipment such as viscometer, Marshall Stability machine and others and the values are as presented. Marshall Stability and flow tests were carried out on the resultant crumb rubber bio asphalt mix produced (Fig. 4, Fig. 5).

## References

[1] Oluwasola E.A., Hainin M.R., Khairul M., Modupe A.E., Idham M.A. (2018). Workability and rheological properties of eva-modified bitumen compared with PG 76 binders. J. Teknol..

[2] Ki O.L., Kurniawan A., Lin C.X., Ju Y.H., Ismadji S. (2013). Bio-oil from cassava peel: a potential renewable energy source. Bioresour. Technol..

[3] Onyango F., Wanjala S.R., Ndege M., Masu L. (2015). Effect of rubber tyre and plastic wastes use in asphalt concrete pavement. World Acad. Sci., Eng. Technol., Int. J. Civil. Environ. Eng..

[4] Metwally Mohamed, Raouf Mohamed Abdel, Williams R. Christopher (2010). Development of Non-petroleum Based Binders for Use in Flexible Pavements.

[5] Zhang R., Wang H., Gao J., You Z., Yang X. (2017). High temperature performance of SBS modified bio-asphalt. Constr. Build. Mater..

[6] Peralta J., Silva H.M., Machado A.V., Williams R.C. (2011). Development of a rubber modified fractionated bio-oil for use as a non-crude petroleum binder. Sem. da Esc. De. Eng..

[7] Peralta J., Williams R.C., Rover M., Silva H.M.R.D.D. (2012). Development of a rubber-modified fractionated bio-oil for use as non-crude petroleum binder in flexible pavements. Transp. Res. Circ..

[8] McNally T. (2011). Polymer Modified Bitumen: Properties and Characterization.

[9] Zhang R., Wang H., You Z., Jiang X., Yang X. (2017). Optimization of bio-asphalt using bio-oil and distilled water. J. Clean. Prod..

