# Supplementary material for: Dataset of mechanical, marshall and rheological properties of crumb rubber – Bio-oil modified hot mix asphalt for sustainable pavement works
Source: Data Brief. 2018 Sep 29;21:63–70. doi: 10.1016/j.dib.2018.09.080 (PMC6187011; doi:10.1016/j.dib.2018.09.080)
Supplement: Supplementary file 1 — Supplementary material [file mmc1.docx]

**CONFLICT OF INTEREST STATEMENT**

For the avoidance of doubt and clarity, this research is part of a B. Eng Thesis of Basorun Adebayo Ofonime, Civil Engineering Department, Landmark University, and there is no specific grant from funding agencies in the public, commercial, or non-profiting sectors.
